# Role of oncology/genetics nurse in management of individuals with hereditary diffuse gastric cancer

**DOI:** 10.1186/1897-4287-10-S2-A70

**Published:** 2012-04-12

**Authors:** M Shanahan, MA Young, G Mitchell

**Affiliations:** 1 Peter MacCallum Cancer Centre, Melbourne, Australia

## Background

Hereditary diffuse gastric cancer (HDGC) is an autosomal dominant, rare familial cancer syndrome in which affected individuals develop diffuse gastric cancer at a young age. Mutations in the E-cadherin gene (CDH1) have a 70% lifetime risk of becoming gastric cancer and women with the gene have a 20-40% risk of developing lobular breast cancer.

## Case study

This paper will report on a series of young adults who have undergone predictive genetic testing and been identified as carrying the CDH1 mutation known to be present in their family. The majority of these patients have been diagnosed with gastric cancer at their first screening gastroscopy.

## Nursing intervention

Individuals who are known to carry the mutation are provided with a personalised cancer risk management plan with the aim of reducing morbidity overwhelmed by the number of appointments they receive and by the variability of information given to them, as care often moves from and between several teams. A care pathway has been developed by the oncology/genetics nurse to ensure optimal communication between the genetics and oncology treatment team, to ensure the best outcome for the patients. The oncology/genetics nurse coordinator with her specialist knowledge of genetics plays a vital role in facilitating ongoing follow up of these individuals, coordinating screening, educating patients and their family on risk management strategies.

## Conclusion

This paper will provide a clinical update on this rare familial syndrome will describe the needs of patients and families affected by this condition and illustrate the contribution that expert nursing care makes to the outcomes of people affected by HDGC.

